# Poster Session I - A133 REAL WORLD EFFICACY OF GLP-2 AGONISTS IN PEDIATRIC INTESTINAL FAILURE: CANADIAN NATIONAL MULTICENTRE CASE SERIES

**DOI:** 10.1093/jcag/gwaf042.133

**Published:** 2026-02-13

**Authors:** A Greaves, S Allam, N Pai, J Griffin, F Chowdhury, A Martinez

**Affiliations:** Pediatric Gastroenterology, BC Children’s Hospital, Vancouver, BC, Canada; University of Toronto Temerty Faculty of Medicine, Toronto, ON, Canada; McMaster University, Hamilton, ON, Canada; University of Manitoba, Winnipeg, MB, Canada; McMaster University, Hamilton, ON, Canada; Pediatric Gastroenterology, BC Children’s Hospital, Vancouver, BC, Canada

## Abstract

**Background:**

Glucagon-like peptide-2 (GLP-2) analogues, particularly teduglutide, have shown promise in enhancing intestinal adaptation and reducing parenteral nutrition (PN) dependence among pediatric patients with short bowel syndrome (SBS). Despite growing clinical use, real-world evidence in children remains limited.

**Aims:**

To evaluate clinical outcomes and institutional experiences with teduglutide therapy in pediatric SBS across multiple Canadian centers.

**Methods:**

A cross-sectional, questionnaire-based study was conducted among pediatric gastroenterology and intestinal rehabilitation programs across Canada between January and September 2025. The survey collected data on patient demographics, indications for teduglutide initiation, duration of therapy, PN requirements before and after treatment, markers of intestinal adaptation, and adverse events.

**Results:**

Data was obtained from five Canadian centers, representing 15 pediatric SBS patients treated with teduglutide. The median age at initiation was 61 months, and the median duration of therapy was 21 months.

**PN outcomes:** 33% achieved complete PN independence, while 53% demonstrated a marked reduction (>/=20% decrease) in PN volume. 7% had minimal response (8.5% reduction) and 7% had no response.

**Intestinal adaptation:** 67% showed improved enteral tolerance and stool consistency.

**Safety:** Reported adverse events included catheter-related infections (20%) and injection-site reactions (7%). No gastrointestinal symptoms, neoplasia, or intestinal obstruction were observed.

Of the 27% with an intact ileocecal valve, 50% achieved complete PN independence. Ethnicity was not included as a factor in the analysis, as the study population was largely homogeneous (∼ 75% of participants from the same racial background), limiting meaningful comparison across groups.

**Conclusions:**

Teduglutide appears to be a valuable adjunct in the management of pediatric SBS within the Canadian context, contributing to reduced PN dependence and improved intestinal adaptation. The therapy was generally well tolerated, with few complications reported. These findings support broader access to teduglutide and highlight the need for standardized protocols and continued multicenter collaboration to optimize outcomes in this population.

**Funding Agencies:**

C-CIRN

